# Electrodeposition of hierarchically structured three-dimensional nickel–iron electrodes for efficient oxygen evolution at high current densities

**DOI:** 10.1038/ncomms7616

**Published:** 2015-03-17

**Authors:** Xunyu Lu, Chuan Zhao

**Affiliations:** 1 School of Chemistry, The University of New South Wales, Sydney, New South Wales 2052, Australia

## Abstract

Large-scale industrial application of electrolytic splitting of water has called for the development of oxygen evolution electrodes that are inexpensive, robust and can deliver large current density (>500 mA cm^−2^) at low applied potentials. Here we show that an efficient oxygen electrode can be developed by electrodepositing amorphous mesoporous nickel–iron composite nanosheets directly onto macroporous nickel foam substrates. The as-prepared oxygen electrode exhibits high catalytic activity towards water oxidation in alkaline solutions, which only requires an overpotential of 200 mV to initiate the reaction, and is capable of delivering current densities of 500 and 1,000 mA cm^−2^ at overpotentials of 240 and 270 mV, respectively. The electrode also shows prolonged stability against bulk water electrolysis at large current. Collectively, the as-prepared three-dimensional structured electrode is the most efficient oxygen evolution electrode in alkaline electrolytes reported to the best of our knowledge, and can potentially be applied for industrial scale water electrolysis.

## Abstract (web summary)

Development of efficient and affordable oxygen evolution catalysts is essential for large-scale electrolytic water splitting. Here, the authors report mesoporous nickel–iron composite nanosheets loaded on macroporous nickel foam substrates, and evaluate their electrocatalytic oxygen evolution in basic media.

## Graphical abstract

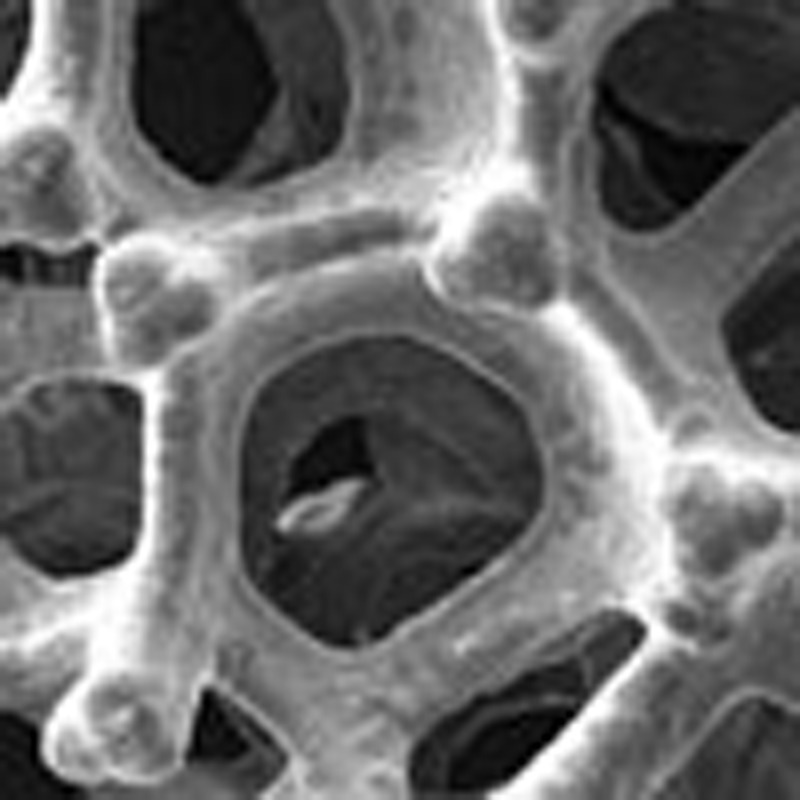

In an electrolytic water splitting cell, the production of H_2_ at the cathode is severely constrained by the sluggish kinetics of the oxygen evolution reaction (OER) on the anode^1^,^2^. As a consequence, highly efficient OER catalysts are required to lower the energy barrier to improve the overall energy efficiency. So far, IrO_2_ and RuO_2_ are the benchmark OER catalysts, owing to their high catalytic activity^3^. Nevertheless, these precious metals are costly and their supply is not sustainable, therefore, are not suitable for large-scale applications. Hence, enormous research efforts have been devoted to the development of low-cost OER electrocatalysts on the basis of first-row transition metals and their oxides such as cobalt phosphate composites, nickel borate composites, cobalt oxide nanoparticles and manganese oxide thin films^4^,^5^,^6^,^7^, which exhibit good OER activity with significantly lowered fabrication costs. Among these catalysts, nickel- and cobalt-based composites have shown promise as active catalyst materials for OER, which usually require overpotentials around 350~450 mV to deliver a current density (*j*) of 10 mA cm^−2^. To further enhance the OER activity of the transition metal-based catalysts to a level that is comparable to the benchmark IrO_2_ and RuO_2_ catalysts, several effective strategies have been reported including downscaling, and/or making nanoporous structures of the catalysts^6^,^8^,^9^,^10^,^11^, using carbon nanomaterials as substrates to immobilize nanoparticle catalysts^12^,^13^ and making multi-metallic catalysts by taking advantage of synergistic metal–metal interactions^14^,^15^,^16^,^17^.

To be used for industrial applications, OER catalysts need to meet more strict criteria such as delivery of very high current densities (≥500 mA cm^−2^) at low overpotentials (≤300 mV), and mechanical robustness and prolonged durability during strong gas evolution^1^,^3^,^18^. Currently, most of the transition metal-based OER composite catalysts reported in literature are powders, which are subsequently coated onto conductive substrates with the aid of polymeric binders, for example, Nafion. The utilization of electrical insulating binders can decrease the contact area between electrolytes and catalytic active sites and deteriorate the electrical conductivity of electrode, leading to receded electrocatalytic performances^19^. The stability of the electrode is also relatively poor and the glued OER catalysts tend to peel off from the substrate especially under high current density and vigorous gas evolution conditions. Electrodeposition is a versatile and scalable industrial process that only requires simple equipment and enables precise control of the nucleation and growth processes, as well as the purity, structures and morphologies of the deposits obtained. Electrodeposition of metal oxides and composite catalysts (for example, NiFe) onto the surface of two-dimensional planar substrates have been reported^20^,^21^,^22^, which, however, usually has limited accessible active sites, as only the few outermost layers are in contact with electrolytes. Furthermore, bubbles generated during OER tend to accumulate on the planar substrates, resulting in significant bubble overpotentials, especially under high current densities and strong gas evolution conditions^23^,^24^,^25^. Fierro and co-workers recently studied the electrodeposition of NiFe onto several metal substrates and found that stainless steel mesh and nickel foam (NF) substrates offer higher activity for OER than nickel foil and mesh^26^. Nevertheless, the metallic NiFe films deposited via galvanostatic reduction of Ni^2+^ and Fe^2+^ are relatively thick (2~8 μm) with high degree of discontinuities, and the electrodes are unstable under long term, large current density operation for water electrolysis^26^.

Herein, we report a hierarchically structured three-dimensional (3D) oxygen electrode prepared via a facile, one-step electrodeposition of amorphous, mesoporous nickel–iron hydroxide (NiFe) nanosheets onto NF substrates. The 3D interconnected bi-continuous macroscopic porous structure across the whole NF substrate offer very high specific surface area, electrical conductivity and integrity^27^,^28^. NiFe is prepared by co-electrodeposition of the Ni and Fe hydroxides onto the NF support, forming ultrathin nanosheets, without use of any binders. The 3D amorphous NiFe/NF is used directly as an oxygen electrode with outstanding OER performance at high current density and low overpotentials.

## Results

### Synthesis and characterization of the NiFe/NF electrode

The electrodeposition of NiFe composites onto NF was undertaken in the electrolyte containing equal molar (3 mM) of nickel (II) and iron (III) nitrates. The deposition potential was controlled at −1.0 V versus Ag/AgCl to reduce NO_3_^−^ ions at the electrode surface to generate hydroxide ions and increase the pH value (equation (1))^29^,^30^. Ni^2+^ and Fe^3+^ ions then reacted with local generated hydroxide ions to form bimetallic hydroxide deposits on the surface of electrodes according to equation (2).


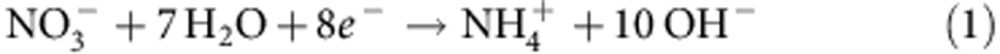



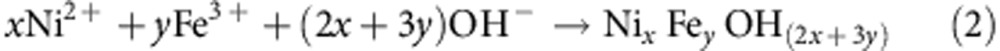


**Figure 1:**
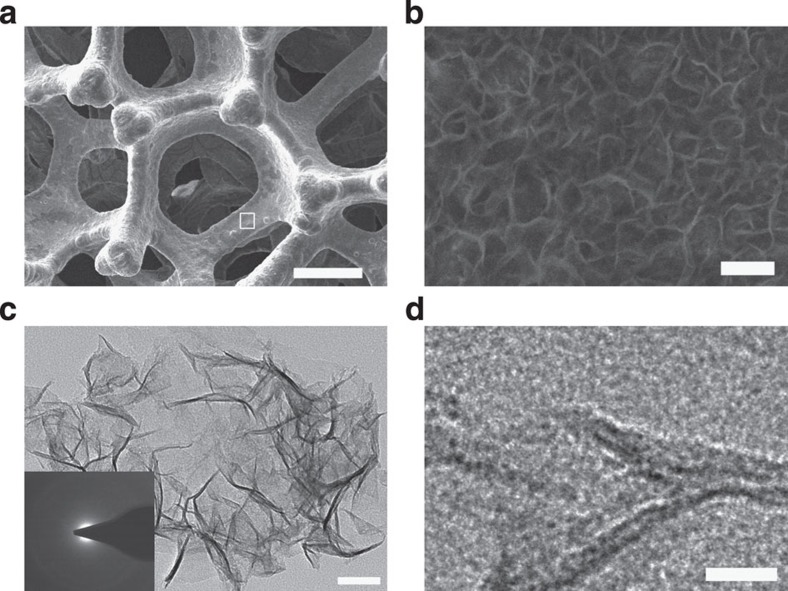
Microscopy measurements of the NiFe/NF electrode. (**a**) SEM image of the NiFe/NF electrode. (**b**) High resolution SEM image of the area squared in **a.** (**c**,**d**) TEM images of NiFe nanosheets scratched off from the NiFe/NF (the inset shows the corresponding selected area diffraction pattern). Scale bars, 200 μm, 200 nm, 50 nm and 10 nm in **a**,**b**,**c** and **d**, respectively.

The electrodeposition process leads to a brown thin film deposited on the NF substrate (Supplementary Fig. 1). Figure 1a shows the scanning electron microscopy (SEM) image of the NiFe/NF composite. The NiFe catalysts are found to be uniformly deposited onto the macroscopic 3D skeleton of NF, resulting in high catalyst loadings as well as large amount of accessible active sites. Figure 1b displays a higher magnification SEM image of the NF surface marked in Fig. 1a. The NiFe deposits show a highly rippled nanosheet structure, which is significantly different from the morphologies of pure NF as well as that obtained when Fe and Ni are deposited individually onto NF (Supplementary Fig. 2). The lateral extension of the nanosheet ranges from 50 nm to several hundred nanometers, and the thickness is generally around 10 nm. The rippled nanosheets are interconnected forming mesoporous NiFe nanostructures, with the pore size around 50 nm.

Figure 1c displays the transmission electron microscopy (TEM) image of the NiFe composites carefully scratched off from the NF substrate. The NiFe nanosheet shows a rippled sheet structure with a dimension around 300 nm, in accordance with the SEM results. The nanosheets are highly transparent, indicating they are ultrathin. High-resolution TEM (Fig. 1d) suggests that the as-prepared NiFe nanosheets are amorphous, with no detection of typical lattice fringes for Ni, Fe or NiFe composites. The inset of Fig. 1c is the selected-area electron diffraction pattern of the nanosheet. The pattern shows a broad and diffused halo ring, further confirming that the as-prepared NiFe nansheets are amorphous^31^. The above results suggest that mesoporous NiFe are formed on macroporous NF forming a hierarchically structured electrode, a configuration that is known to be beneficial for electrocatalytic gas evolution.

The composition of the NiFe deposits was determined by X-ray photoelectron spectroscopy (XPS). As displayed in Supplementary Fig. 3a, Ni, Fe as well as O are detected in the XPS spectrum, suggesting that a bimetallic composite is obtained. The Ni 2*p* spectrum (Supplementary Fig. 3b) can be fitted into two spin-orbit peaks, namely Ni 2*p*_1/2_ and Ni 2*p*_3/2_ at 874 and 856 eV, with two shakeup satellites, indicating that Ni is in Ni^2+^ oxidation state^32^. Supplementary Figure 3c exhibits the high resolution Fe 2*p* spectrum. The observation of Fe 2*p*_1/2_ and Fe 2*p*_3/2_ at ~725 and ~712 eV with a shakeup satellite at ~720 eV confirms that Fe is mostly in Fe^3+^ oxidation state in the NiFe composite^33^. The atomic ratio of Ni and Fe in the composite deposited is determined to be 3 by taking the average of the XPS results obtained on the NiFe-coated Pt electrode at four different points (see Methods for experimental details). Hence, the NiFe composite obtained is specified as Ni_3_Fe(OH)_9_.

**Figure 2:**
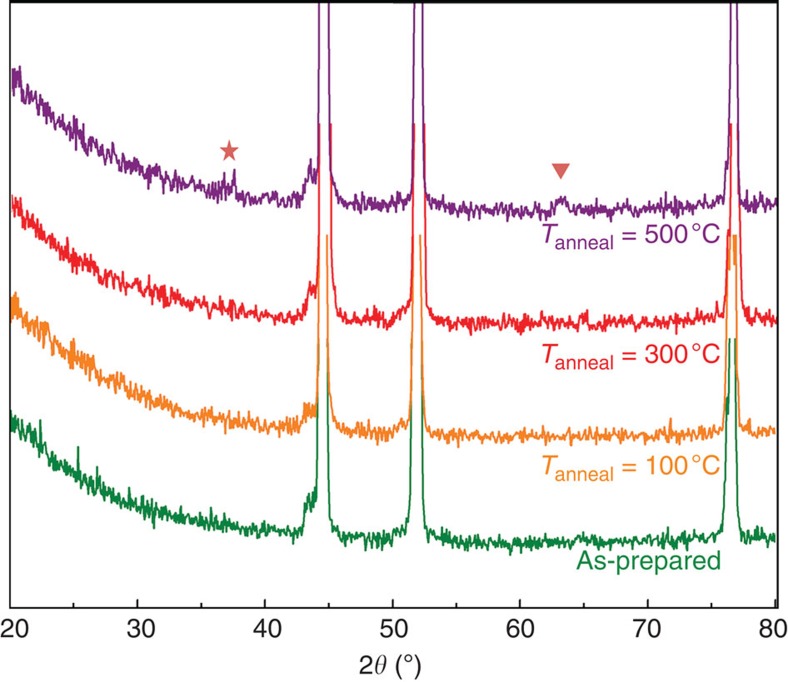
XRD patterns of as-prepared and annealed NiFe/NF samples. The pentagram and triangle represent the Bragg reflections for hematite^1^.

Supplementary Fig. 4 shows the elemental distributions of Ni and Fe in the NiFe/NF composite detected by energy-dispersive X-ray spectroscopy. The Ni and Fe are indexed with red and green colour in the energy-dispersive X-ray spectroscopy images, respectively. Both Ni and Fe are found to distribute uniformly in the whole area tested, confirming the deposition of homogeneous NiFe bimetallic composites over the NF substrate. The XRD pattern shown in Fig. 2 exhibits only three diffraction peaks of the NF at 44.5°, 51.8° and 76.4°, respectively^34^, without the detection of any new diffraction peaks, further confirming that the NiFe deposited onto NF is amorphous in nature. Post-annealing treatment will endow NiFe/NF crystallinity. Also shown in Fig. 2, at the annealing temperature (*T*_anneal_) ≥500 °C, new diffraction peaks at 36.5° and 63.5° are emerged, ascribed to the crystalline hematite structure^1^. However, the OER catalytic activity of NiFe/NF decreases accordingly with the increased *T*_anneal_ (Supplementary Fig. 5), indicating that the amorphous structures of NiFe composite are more active for OER. This result is in accordance with that observed by Smith *et al*.^1^, and could possibly be ascribed to the larger surface area and higher electrical conductivity that are offered by amorphous phase materials^31^.

### Electrocatalytic oxygen evolution at the NiFe/NF electrode

**Figure 3:**
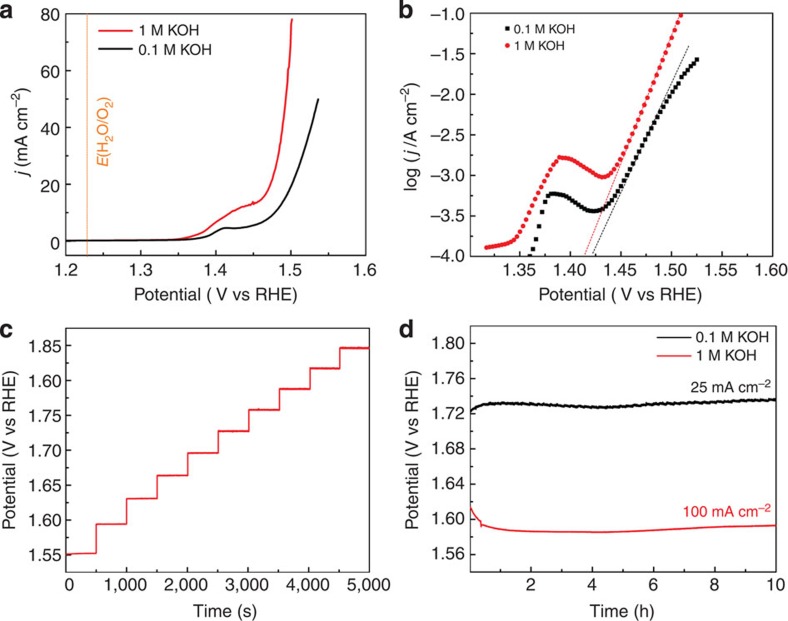
Electrochemical characterizations of the NiFe/NF oxygen electrode. (**a**) OER polarization curves of the NiFe/NF oxygen electrode in 0.1 and 1 M KOH solutions at 5 mV s^−1^ with 95% *iR*-compensations. (**b**) Tafel plots of the NiFe/NF oxygen electrode in 0.1 and 1 M KOH at 0.1 mV s^−1^ with 95% *iR* compensation. (**c**) Multi-current process obtained with the NiFe/NF oxygen electrode in 1 M KOH. The current density started at 50 mA cm^−2^ and finished at 500 mA cm^−2^, with an increment of 50 mA cm^−2^ every 500 s. (**d**) Chronopotentiometric curves obtained with the NiFe/NF oxygen electrode in 0.1 and 1 M KOH with constant current densities of 25 and 100 mA cm^−2^, respectively. vs, versus.

The electrocatalytic performances of the NiFe/NF electrode for OER in alkaline media are studied and shown in Fig. 3. All the data were recorded in a standard three-electrode electrochemical cell using the NiFe/NF as the working electrode, a Ag/AgCl (3 M KCl) as the reference electrode and a Pt plate as the counter electrode. Potentials obtained in this study were all calibrated to the reversible hydrogen electrode (RHE) reference for the comparison purpose (see Methods for details).

The OER processes of the NiFe/NF electrode in 0.1 and 1 M KOH exhibit the same onset potential of 1.44 V. At higher potentials, the more rapid increase in current is observed for 1 M KOH owing to higher conductivity of the electrolyte. At the same overpotential (*η*)=270 mV, *j*=80 mA cm^−2^ is obtained in 1 M KOH solution, while 20 mA cm^−2^ is obtained in 0.1 M KOH. In comparison, the current obtained from the bare NF substrate, and NF substrates electrodeposited with Ni or Fe individually, exhibit significantly inferior OER activity (Supplementary Fig. 6), suggesting the high catalytic activity is originated from the NiFe composites, and the synergistic effect between Ni and Fe in the composites^35^. The small oxidation process detected at 1.41 V before the onset of OER is corresponding to the formation of Ni (III) or Ni (IV) species, which are the active sites to catalyse OER^35^. Moreover, the OER performances of the NiFe/NF are evaluated by Tafel equation *η*=*b* × log (*j*/*j*_*0*_), where *b* is the Tafel slope and *j*_*0*_ is the exchange current density. As shown in Fig. 3b, the slopes remain linear even at high values of *j*, indicating fast electron and mass transfer between the catalyst and the electrolyte. The Tafel slope of NiFe/NF in 0.1 and 1 M KOH are 33 and 28 mV dec^−1^, respectively, which are even lower than the benchmark IrO_2_ and RuO_2_ catalysts^36^,^37^,^38^.

Figure 3c represents a multi-step chronopotentiometric curve obtained at NiFe/NF in 1 M KOH. In the experiment, the current is increased from 50 to 500 mA cm^−2^ with an increment of 50 mA cm^−2^ per 500 s, and the corresponding changes of potential are recorded. At the start of 50 mA cm^−2^, the potential immediately levels off at 1.55 V, and remains constant for the rest 500 s. Similar results are obtained for all current densities tested herein up to 500 mA cm^−2^. These chronopotentiometric responses reflect the excellent mass transport properties (inward diffusion of OH^−^ and outwards diffusion of oxygen bubbles), conductivity and mechanical robustness of the NiFe/NF electrode.

The electrochemical stability of the NiFe/NF electrode is tested in bulk electrolysis of water, as displayed in Fig. 3d. In 0.1 M KOH, the potential required to deliver a *j* of 25 mA cm^−2^ is ~1.73 V, and then stabilizes around this value during the 10 h reaction session, with very small voltage fluctuations (<10 mV). The NiFe/NF electrode works more efficiently in 1 M KOH. The potential required to deliver a *j* of 100 mA cm^−2^ is ~1.60 V, and also remains stable during the 10 h electrolysis. In contrast, the OER catalytic activity of NF substrate alone decays gradually in prolonged bulk water electrolysis (Supplementary Fig. 7), due to the surface passivation by the formation of NiO_x_ layers^39^. Although the gas evolution at the NiFe/NF electrode during the electrolysis is very vigorous, gas bubbles dissipate rapidly into the solution with no bubble accumulation observed on the electrode surface. The outstanding physical stability of NiFe/NF is also confirmed by SEM. Supplementary Figure 8 shows the high resolution SEM image of NiFe/NF after >100 h of bulk water electrolysis. As shown in the image, the porous morphology of the NiFe nanosheets is well-preserved, and no detachment or dissolution of the catalyst from the NF substrate is detected.

The performance of NiFe/NF is futher compared with other state-of-the-art electrocatalysts in alkaline media. Supplementary Table 1 summarizes the overpotentials required to deliver a *j* of 10 mA cm^−2^, a value relative to solar fuel synthesis because this current density roughly matches the spectrum for a 10% efficient solar-to-fuel device^40^,^41^. The overpotential of the NiFe/NF electrode outperforms all the electrocatalysts reported, including the benchmark Ir/C and Ru/C electrocatalysts^4^. Furthermore, the OER activity of the as-prepared NiFe/NF electrode is also significantly higher than previously reported NiFe-based electrodes^3^,^6^,^26^. For example, as shown in Supplementary Fig. 9, in 30 wt% KOH electrolyte, the overpotentials for obtaining current densities of 100 and 300 mA cm^−2^, and the Tafel slope obtained with our NiFe/NF electrodes are 270 mV, 340 mV and 32 mV dec^−1^, respectively, which are 39 mV, 61 mV and 8 mV dec^−1^ lower than the best NiFe/NF electrode reported previously under the same conditions^26^. Our electrodes also exhibit outstanding long-term stability, while significant detachment and shrinking of NiFe films were observed for the electrode prepared by Pérez-Alonso *et al*.^26^ for long-term bulk water electrolysis.

Furthermore, the superior OER catalytic activity of NiFe/NF is evaluated by using both the electrochemical surface area (ECSA) and geometric surface area (GSA). The ECSA is calculated on the basis of the measured double-layer capacitance of the NiFe/NF electrode in 1 M KOH according to the method established previously^42^ and the results are shown in Supplementary Fig. 10. A roughness factor of 50 is obtained with the NiFe/NF electrode (see details in Supplementary Methods). Therefore, the current density on the basis of ECSA (*j*_ECSA_) is simply calculated by dividing the current density obtained with GSA (*j*_GSA_) by the roughness factor. As shown in Supplementary Table 2, even at a lower overpotential of 300 mV, NiFe/NF exhibits a significantly higher catalytic activity compared with IrO_x_, NiCoO_x_ and NiFeO_x_ (ref. 42), as exemplified by much higher current densities. Collectively, the data suggest that the as-prepared NiFe/NF is the most active OER electrocatalyst in alkaline electrolytes reported so far.

**Figure 4:**
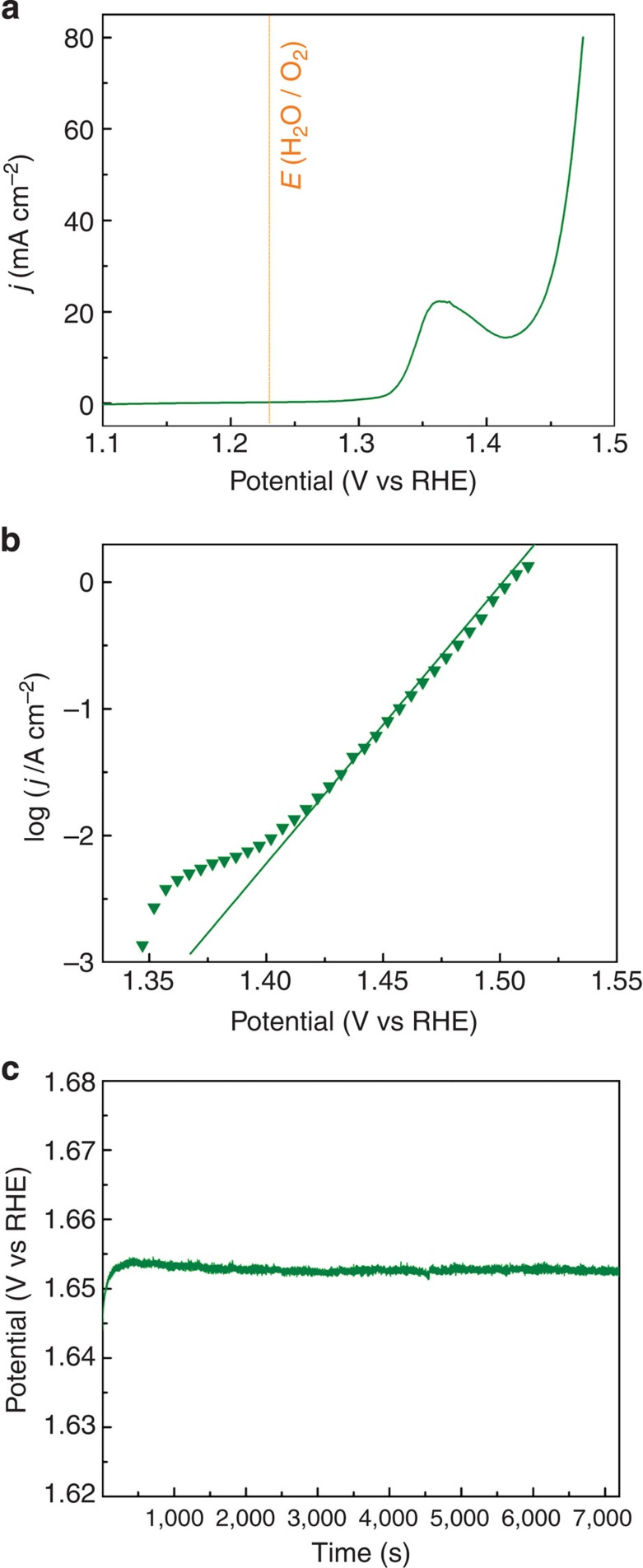
Electrochemical performances of the NiFe/NF electrode in 10 M KOH. (**a**) The OER polarization curve of NiFe/NF in 10 M KOH at 5 mV cm^−1^ with 75% *iR* compensation. (**b**) The Tafel plot of NiFe/NF in 10 M KOH at 0.1 mV s^−1^ with 95% *iR* compensation. (**c**) The chronopotentiometry curve of NiFe/NF in 10 M KOH with a constant current density of 500 mA cm^−2^. vs, versus.

Finally, we tested the catalytic performances of the NiFe/NF electrode under extreme conditions such as concentrated KOH solution and very high current density, which are often encountered in commercial alkaline water electrolysers^43^. Figure 4a represents the OER polarization curve of NiFe/NF in 10 M KOH. The OER starts at an onset potential of 1.41 V, and a *j*=80 mA cm^−2^ obtained at 1.47 V, 60 and 30 mV lower than that obtained in 0.1 and 1 M KOH, respectively. The Tafel plot (Fig. 4b) exhibits excellent linearity, even at *j* as high as 1,000 mA cm^−2^, attributed to the high conductivity of the supporting NF substrates as well as the electrolytes. The overpotentials to deliver *j* of 500 and 1,000 mA cm^−2^, are merely 240 and 270 mV, respectively, according to the Tafel plot. These results faithfully satisfy the requirements for commercial water electrolysers (for example, *j*≥500 mA cm^−2^ at *η*≤300 mV)^1^. Furthermore, the stability of NiFe/NF in 10 M KOH is also investigated in bulk electrolysis at *j*=500 mA cm^−2^. The electrodes exhibit exceptional stability during the whole session tested herein (Fig. 4c), further indicating that the NiFe/NF electrode is catalytically stable and mechanically robust.

## Discussion

The performance of the NiFe/NF electrodes can be attributed to several factors: (i) intrinsically high activity of the NiFe nanosheet catalysts, (ii) unique hierarchically structured porous configuration, which enables large working surface area and excellent gas bubble dissipation ability; (iii) low electrical resistance of the whole water splitting cell by using the binder-free electrodeposition approach and high concentration of electrolytes.

**Figure 5:**
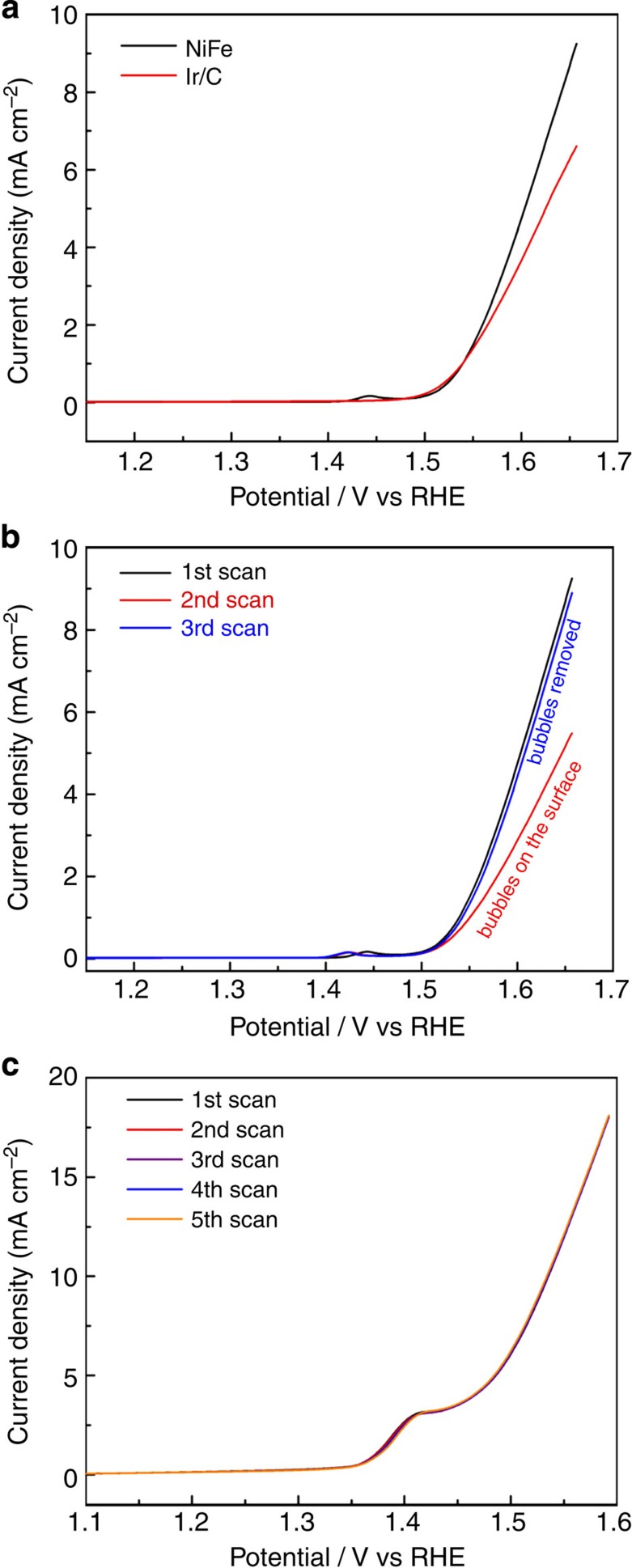
Contributing factors to the high activity of the NiFe/NF electrode. (**a**) The first OER polarization curves obtained with the NiFe- and Ir/C-coated GC electrodes, respectively. (**b**) The first three OER polarization curves obtained with the NiFe deposited onto GC electrodes. (**c**) Five consecutive polarization scans obtained with the NiFe/NF electrode. All the measurements were carried out in a 0.1 M KOH solution at a scan rate of 5 mV s^−1^. vs, versus.

As mentioned above, the overpotential required to initiate the OER process at the NiFe/NF electrode is merely ~200 mV, which is the lowest among non-precious metal-based catalysts reported in literature, indicating significantly diminished OER activation energies. The OER activity of NiFe is even higher than the benchmark Ir catalysts in alkaline media. Figure 5a displays the OER polarization curves obtained with NiFe electrodeposited and Ir/C (20 wt% of Ir, Premetek Co.) drop-casted GC electrodes, respectively, in 0.1 M KOH solution. Even though having an identical onset OER potential, NiFe exhibits significantly higher catalytic activity at elevated overpotentials. At an overpotential of 400 mV, the *j* obtained with NiFe is ~30% higher than the Ir/C. The intrinsic OER catalytic activities of NiFe and Ir/C are further evaluated by calculating the turnover frequency (TOF) assuming that all the Ni and Ir sites are involved in OER. The amount of NiFe catalyst electrodeposited is determined to be 32 μg cm^−2^ as using a electrochemical quartz crystal microbalance (EQCM, see details in Methods). The TOF associated with NiFe at the overpotential of 400 mV is 0.075 s^−1^, which is almost threefold higher than that obtained with Ir/C (0.027 s^−1^). These collective data confirm that the as-deposited NiFe is highly efficient towards OER.

The performance is also strongly related to the hierarchically structured mesoporous NiFe catalyst on the macroporous NF frame, which provides abundant active sites to be exposed to electrolytes. The attachment of gas bubbles on the surface of electrodes during OER, however, can potentially block the active sites of the catalysts and prohibits ionic transportation, and is one of the major sources of overpotential. This is especially an issue for highly active electrodes under high current density operation, as more bubbles will be generated owing to the faster reaction kinetics^25^,^44^. The hierarchical porous structure of the NiFe/NF electrode is found to be highly effective in dissipating the gas bubbles. Figure 5b displays the three consequent OER polarization curves obtained with the NiFe electrodeposited onto the planar GC electrode in 0.1 M KOH solution. The second polarization curve is severely affected by the gas bubbles generated on the first scan, exhibiting a drastic decrease in the OER current. Only when the bubbles attached on NiFe/GC electrode are carefully removed by thorough water rinsing and subsequent nitrogen blowing, can the catalytic activity of the NiFe/GC electrode be recovered (third scan, Fig. 5b). In contrast, five consequent OER polarization curves obtained with the 3D NiFe/NF electrode under the same conditions exhibit almost no decrease in OER activity (Fig. 5c) suggesting very minor impact of gas bubble on the performance. This is also confirmed in high current density (100 mA cm^−2^) bulk water electrolysis (10 h), where no gas accumulation on the electrode surface and no voltage drop are observed (Fig. 4c). The superior gas dissipation ability can possibly be arisen from two levels: (i) the interconnected NiFe nanosheets form hierarchical mesopores (~50–100 nm), which is known to improve the wetting properties of the electrode surface and facilitates the detachment of bubbles^24^,^25^; (ii) the macroporous NF (pore size ranges from 100 to 200 μm) enables a fast dissipation of the large oxygen bubbles into the electrolyte. All these features contribute to the highly diffusive NiFe/NF gas anode.

Finally, the binder-free, electrodeposition approach produces firmly bonded NiFe composites on the highly conductive NF, which minimizes the resistance arisen from the contact between catalysts and NF substrates. The application of polymeric binders (for example, Nafion) for powder-based catalysts tends to impede the charge transport during catalytic reactions^19^, and also deteriorate the mechanical stability of the catalysts under high current operation. Application of high concentration electrolytes provides further reduction of the whole cell resistance and overpotentials (Supplementary Fig. 11), provided that the catalysts remain stable, as is the case of NiFe/NF electrodes.

In summary, a highly efficient, freestanding oxygen evolution electrode is prepared via electrodeposition of mesoporous amorphous NiFe hydroxide nanosheets onto macroporous NF substrates without using chemical binders. The as-prepared NiFe/NF electrode has hierarchical micro- to nanoscale porosities, which offer large active surface area, fast mass transport and fast electron transport in the electrode. In alkaline electrolytes, NiFe/NF catalyses OER at very low overpotentials (~200 mV) with prominent durability under high current densities. The highest catalytic activity of NiFe/NF is obtained in 10 M KOH to deliver a *j* of 500 mA cm^−2^ at an overpotential of 240 mV. It is anticipated that the facile, one-step electrodeposition approach can be easily adapted in industry for large-scale production.

## Methods

### Preparation of NiFe/NF

NF (thickness: 1.6 mm, bulk density: 0.45 g cm^−3^, Goodfellow) was sonicated in 5 M HCl solution for 20 min to remove the NiO_*x*_ layer on the surface, and rinsed subsequently with water and ethanol, then dried in air. The electrodeposition was carried out in a standard three-electrode electrochemical cell containing NF as the working electrode, a parallel positioned platinum plate as the auxiliary electrode and a Ag/AgCl (3 M KCl) as the reference electrode. The electrolyte bath contained 3 mM Ni(NO_3_)_2_·6H_2_O and 3 mM Fe(NO_3_)_3_·9H_2_O. To optimize the compositions of the NiFe deposit, the total moles of Ni^2+^ and Fe^3+^ in the electrolyte were maintained at 6 mM while the molar ratio of Ni^2+^ and Fe^3+^ systematically varied. We found that a 1:1 molar ratio of Ni^2+^ and Fe^3+^ yields the NiFe/NF with the highest OER catalytic activity. The constant potential electrodeposition was then carried out at −1.0 V (versus Ag/AgCl) at 10°C. The optimized deposition time of NiFe has been determined to be 300 s. Short deposition time results in insufficient active sites, whereas prolonged deposition time leads to thick composite films and can inhibit the charge transfer between NiFe and NF, which both deteriorate the catalytic performances of the electrodes obtained. After deposition, the NF was carefully withdrawn from the electrolyte, rinsed with water and ethanol, then sonicated briefly in ethanol and left dry in air. Besides NF, NiFe composites were also electrodeposited onto GC (0.07 cm^2^) and Pt (0.196 cm^2^) electrodes following the same procedures. To prepare the Ir/C-coated GC electrode, 5 mg of Ir/C (20 wt% of Ir, Premetek Co.) was dispersed in 1 ml of water and ethanol solution (1:1, v/v), followed by the addition of 25 μl of Nafion 117 solution (Sigma-Aldrich). The mixture was then sonicated briefly to form a homogenous ink. Three microlitres of the ink was drop-casted onto the surface of the 0.07 cm^2^ GC electrode and left dried in air. The amount of Ir loaded onto GC electrode was 40 μg cm^−2^.

### Physical characterization

XPS was performed on a Thermo ESCALAB250i X-ray Photoelectron Spectrometer. SEM was carried out using a FEI Nova NanoSEM 230 with a 10 kV accelerating voltage. TEM was performed using a Philips CM 200 microscope. XRD was performed on a PANalytical X’Pert instrument.

### Electrochemical characterization

All electrochemical measurements were carried out with a CHI 760 electrochemical workstation. As-prepared NiFe/GC or NiFe/NF were used directly as the working electrode without further treatments. A Pt plate or a graphite rod was used as the counter electrode and was separated from the working electrode chamber using a ceramic frit. All potentials measured were calibrated to RHE using the following equation: *E*_RHE_=*E*_Ag/AgCl_+0.197 V+0.059 pH. OER polarization curves were recorded at a scan rate of 5 mV s^−1^. Before recording, the potential of NiFe/NF was scanned for five cycles in KOH solutions until a stable cyclic voltammogram was recorded. Tafel slopes were derived from OER polarization curves obtained at 0.1 mV s^−1^ and 95% *iR* compensation in all the three KOH solutions using NiFe/NF as the working electrode. Chronopotentiometric and chronoampermetric measurements were obtained under the same experimental setup without compensating *iR* drop.

### Electrochemical quartz crystal microbalance

EQCM measurements were performed on a CHI 440C time-resolved EQCM (CH Instruments) with a three-electrode configuration. An AT-cut platinum-coated quartz crystal of 7.995 MHz resonance frequency with the geometrical area of 0.196 cm^2^ was used as the substrate with platinum wire and Ag/AgCl (3 M KCl) as respective counter and reference electrodes. An aqueous solution containing 3 mM of Ni(NO_3_)_2_·6H_2_O and 3 mM of Fe(NO_3_)_3_·9H_2_O was used as the electrolyte. The electrodeposition was performed at 10 °C in potentiostatic mode at −1.0 V versus Ag/AgCl for 300 s and the corresponding change in resonance frequency measured. The change in mass per unit area, Δ*m*, was calculated from the changes in resonance frequency, Δ*f*, using the Sauerbrey equation^45^: 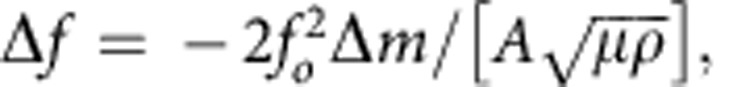
where *f*_*o*_ is the resonant frequency of the quartz resonator, *A* is the area of the platinum coated onto the crystal, *μ* is the shear modulus of the quartz (2.947 × 10^11^ g cm^−1^ s^−2^) and *ρ* is density of the quartz (2.648 g cm^−3^).

### Calculation of turnover frequency

The TOF values of NiFe and Ir/C coated on GC electrodes were calculated according to equation^6^,^13^: TOF=*j* × *A/*(4 × *F* × *m*), where *j* is the current density obtained at overpotential of 400 mV in A cm^−2^, *A* is the surface area of the GC electrode (0.07 cm^−2^), *F* is the Faraday efficiency (96,485 C mol^−1^) and *m* is the number of moles of the Ni and Ir deposited onto the GC electrodes.

## Author contributions

C.Z. directed the project. X.L. performed the experiments. C.Z. and X.L. planned the experiments, analysed the data and co-wrote the paper.

## Additional information

**How to cite this article:** Lu, X. *et al*. Electrodeposition of hierarchically structured three-dimensional nickel–iron electrodes for efficient oxygen evolution at high current densities. *Nat. Commun*. 6:6616 doi: 10.1038/ncomms7616 (2015).

## Supplementary Material

Supplementary Information Supplementary Figures 1-11, Supplementary Tables 1-2, Supplementary Methods and Supplementary References
