# Supplementary material for: Electrodeposition of hierarchically structured three-dimensional nickel–iron electrodes for efficient oxygen evolution at high current densities
Source: Nat Commun. 2015 Mar 17;6:6616. doi: 10.1038/ncomms7616 (PMC4382694; doi:10.1038/ncomms7616)
Supplement: Supplementary Information — Supplementary Figures 1-11, Supplementary Tables 1-2, Supplementary Methods and Supplementary References [file ncomms7616-s1.pdf]

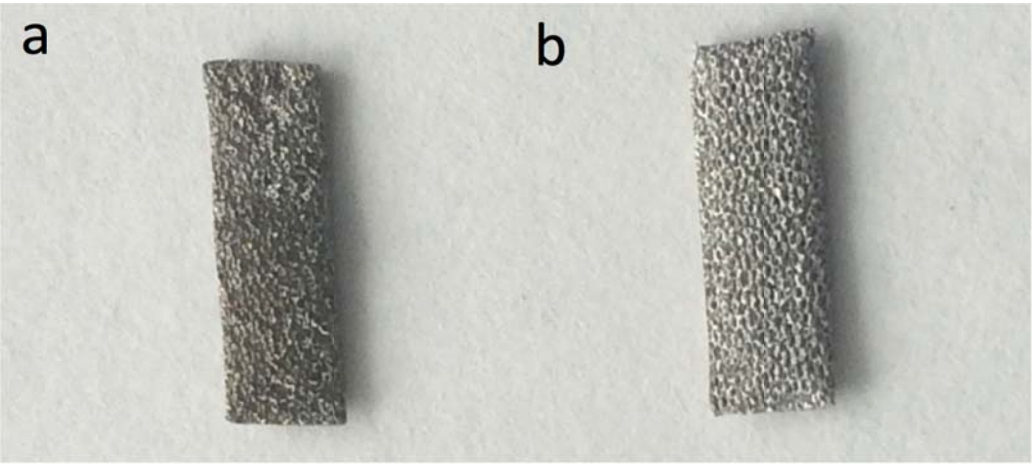

Supplementary Figure 1. Photographs of **(a)** the NiFe/NF electrode and **(b)** the NF substrate.

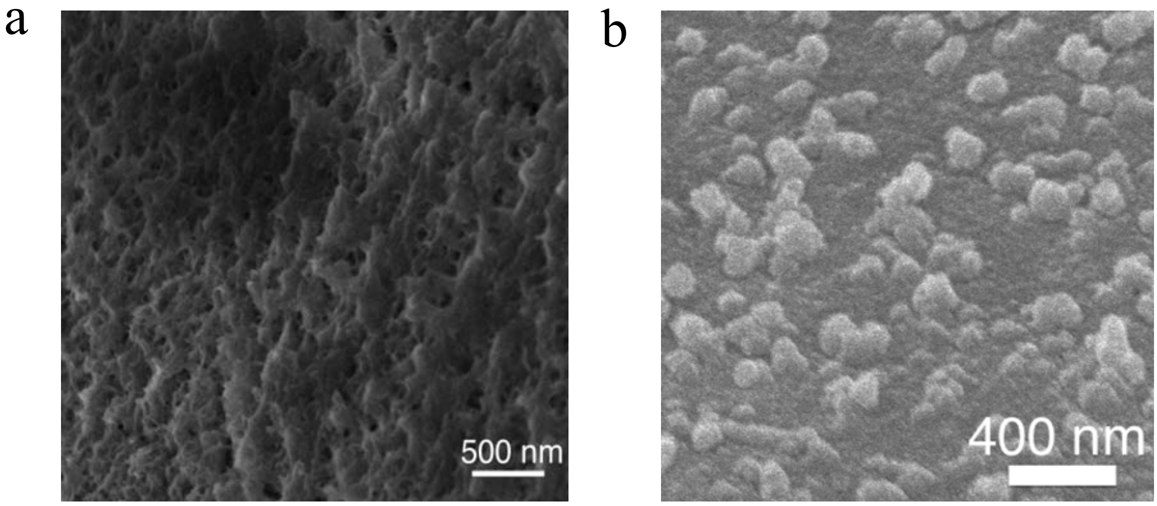

Supplementary Figure 2. SEM images of **(a)** iron and **(b)** nickel deposited on the surface of nickel foam substrates.

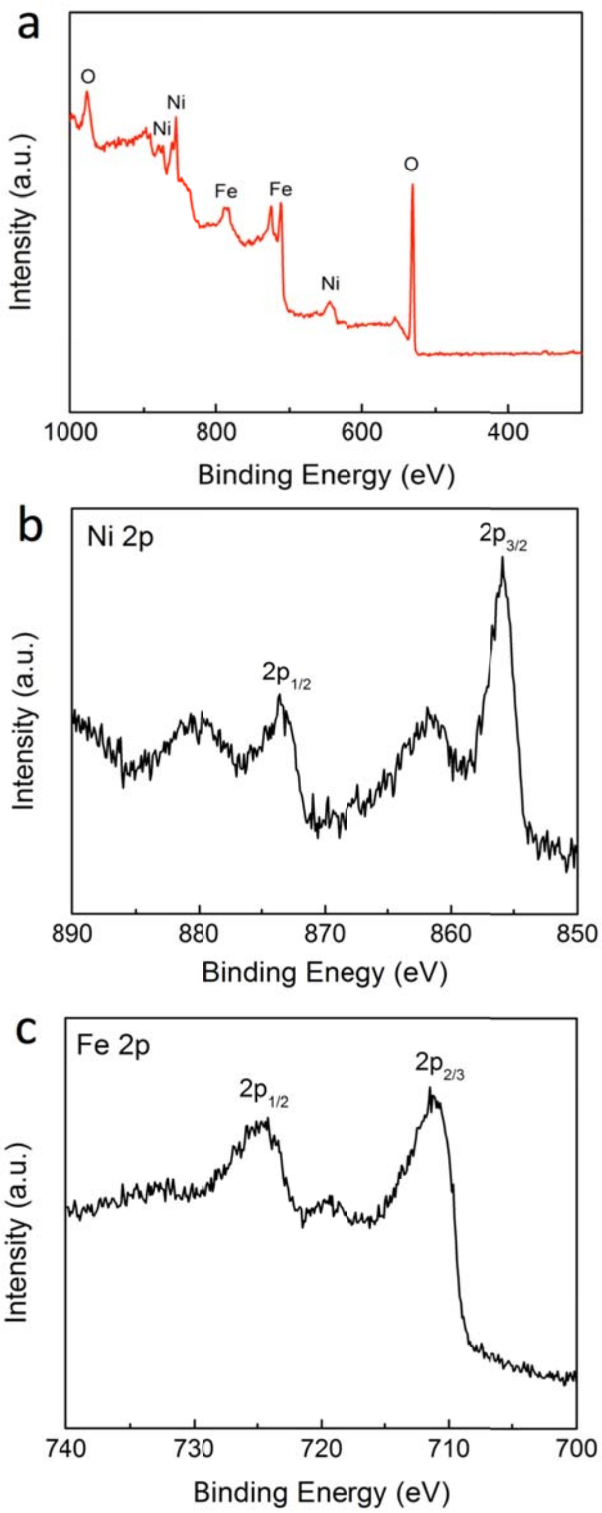

Supplementary Figure 3. (a) XPS survey spectra of the NiFe composites deposited on Pt electrode. **(b, c)** High resolution XPS spectra of Ni 2p and Fe 2p, respectively.

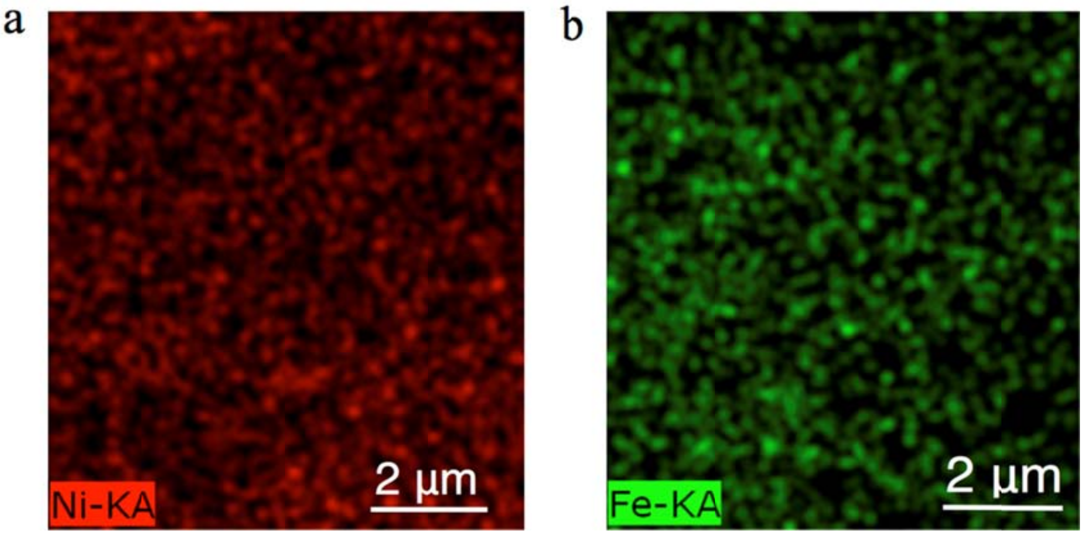

Supplementary Figure 4. EDX mapping of NiFe/NF obtained with SEM, **(a)** nickel and **(b)** iron. The red color indicates the presents of nickel, while the green color represents iron.

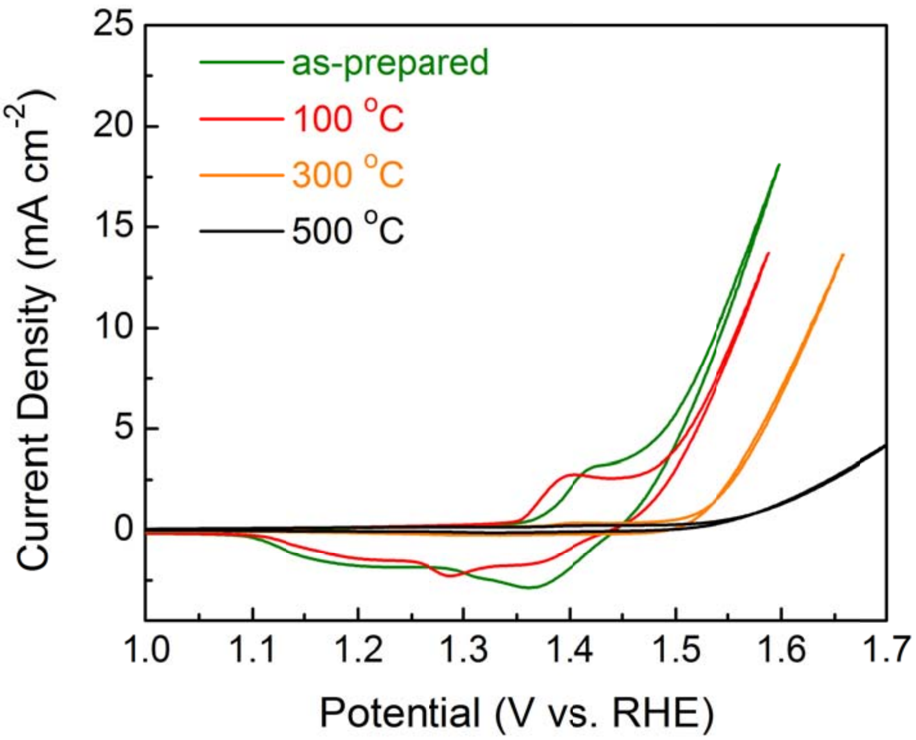

Supplementary Figure 5. Cyclic voltammograms (CVs) obtained with NiFe/NF treated with different annealing temperatures. The CVs were recoreded in a 0.1 M KOH solution at a scan rate of 5 mV s^{-1}.

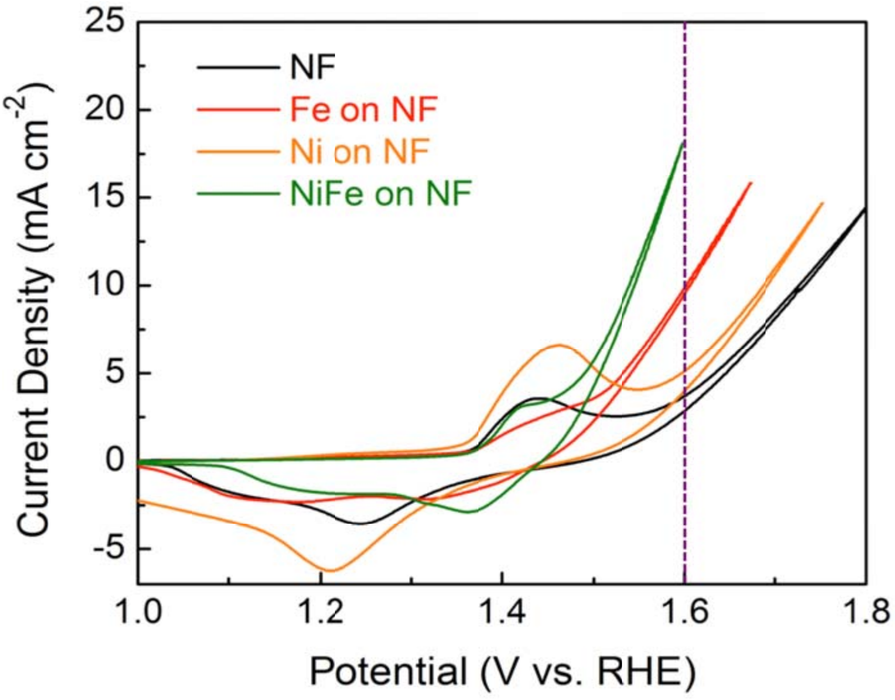

Supplementary Figure 6. CVs obtained with pure NF, Fe/NF, Ni/NF and NiFe/NF in 0.1 M KOH at 5 mV s^{-1}, respectively.

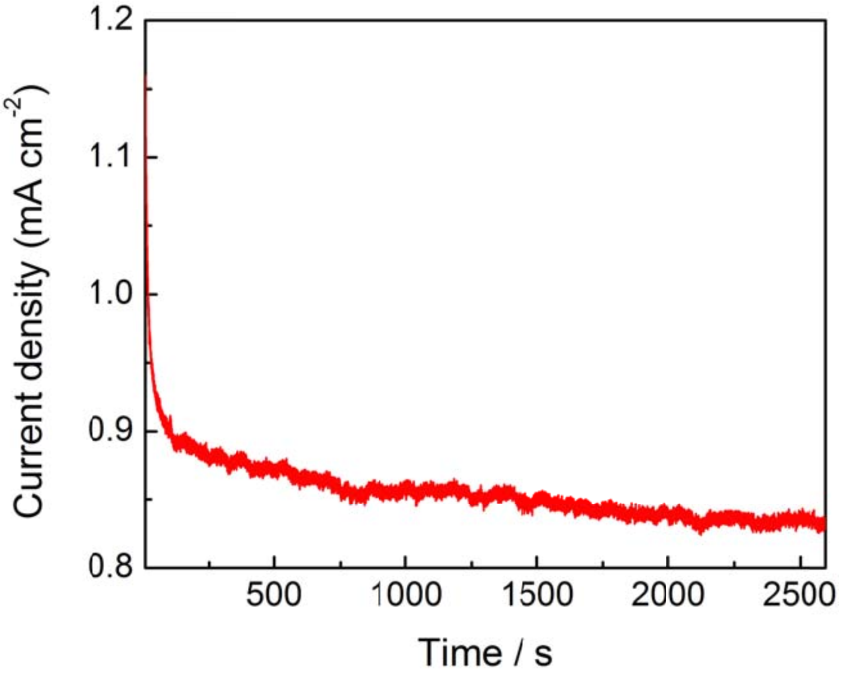

Supplementary Figure 7. The chronoampemetric curve obtained with pure NF substrate in 1 M KOH solution at 1.48 V vs. RHE.

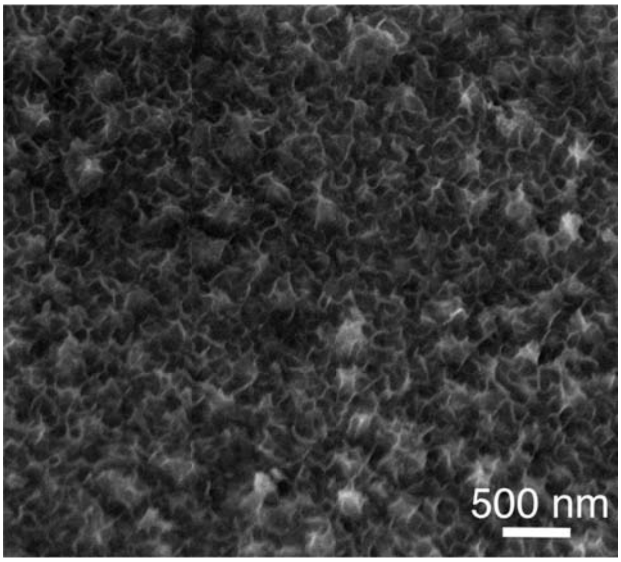

Supplementary Figure 8. SEM image of NiFe/NF after long-term of bulk water electrolysis (> 100 h).

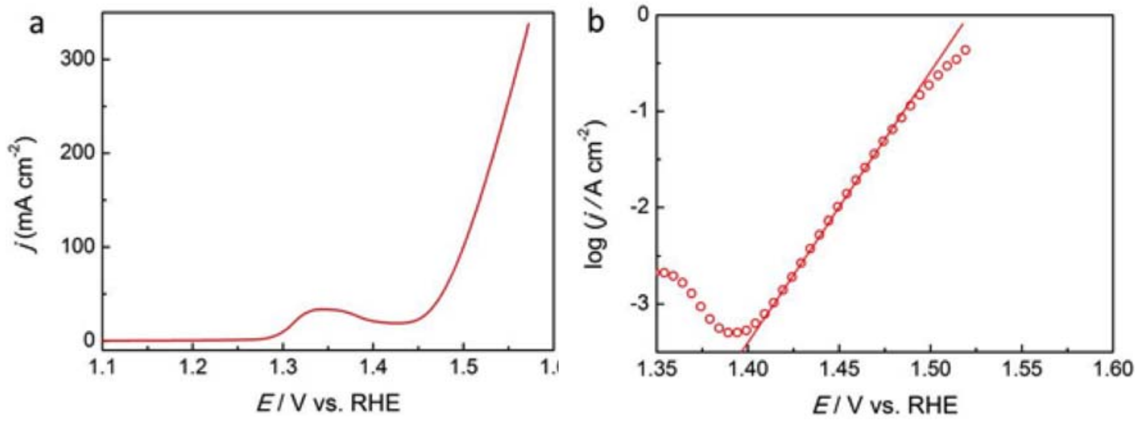

Supplementary Figure 9. a, The OER polarization curve obtained with the NiFe/NF electrode in 30 wt% KOH at 5 mV cm^{-1} without *iR* compensation. **b,** The Tafel plot obtained with the NiFe/NF electrode in 30 wt% KOH at 0.1 mV s^{-1} with 95% *iR* compensation.

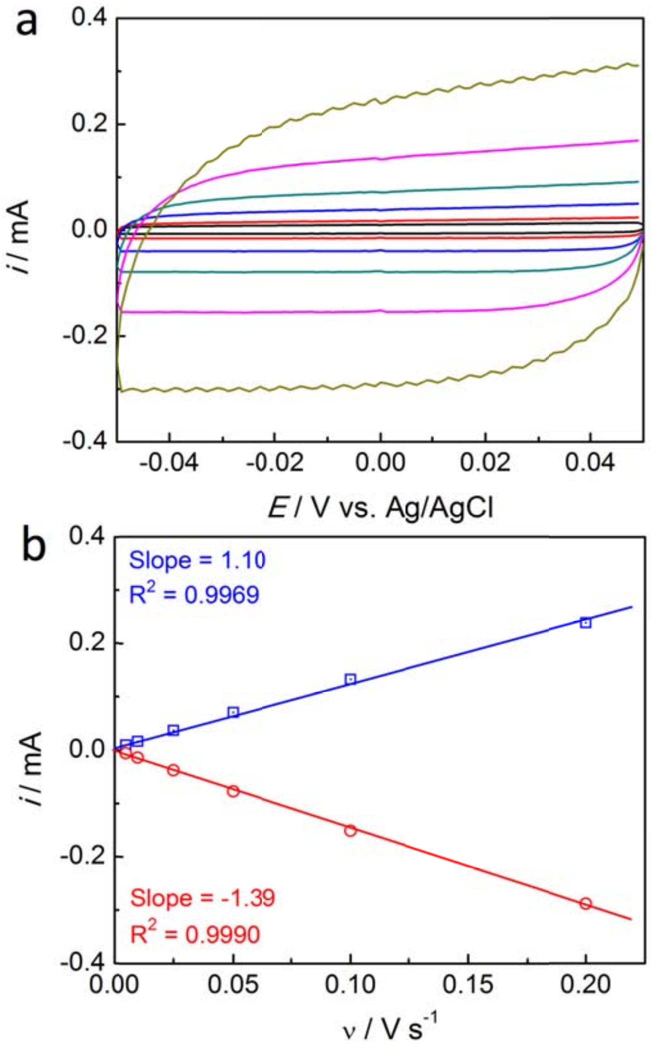

Supplementary Figure 10. (a) Charging currents measured in the non-Faradaic potential range of -0.05 V to 0.05V at scan rates of 5, 10, 25, 50, 100, 200 and 400 mV s^{-1}, respectively. **(b)** The cathodic (red circle) and anodic (blue square) charging currents measured at 0 V vs Ag/AgCl, plotted against the scan rates. The double-layer capacitance determined from this system is taken by the average of the absolute value of anodic and cathodic slopes of the linear fits.

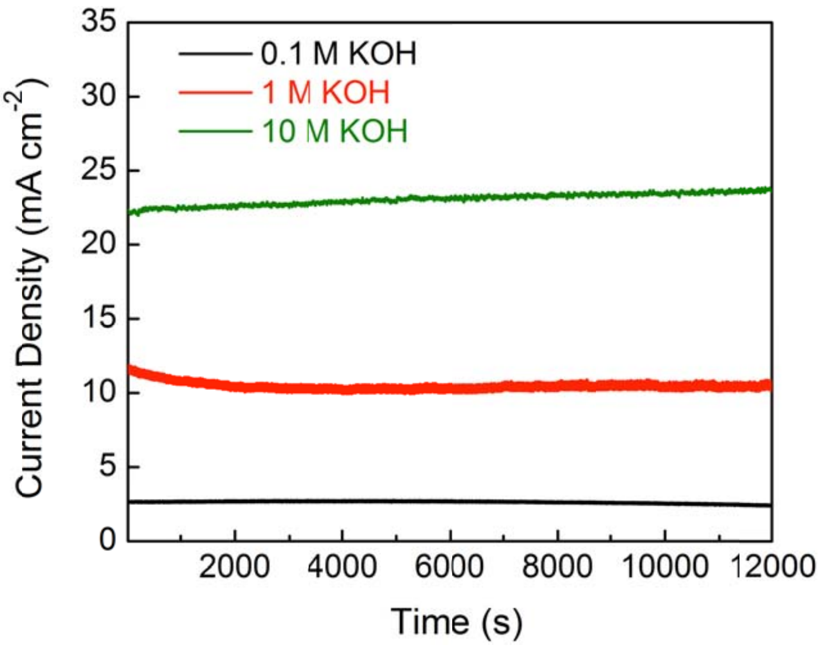

Supplementary Figure 11. Chronoampemetric curves of the NiFe/NF electrode in 0.1, 1 and 10 M KOH with a constant overpotential of 250 mV.

Supplementary Table 1 OER activities of some benchmark electrocatalysts in alkaline solutions with a current density of 10 mA cm^{-2}

Materials	Electrolyte	η/mV	Reference
NiFe/NF	0.1 M KOH	240	This work
NiFe/NF	1 M KOH	215	This work*
Co_3O_4	1 M KOH	328	1
Co_3O_4/Graphene	1 M KOH	310	2
$Ni_{0.9}Fe_{0.1}O_x$	1 M KOH	336	3
20 wt% Ir/C	0.1 M KOH	380	4
20 wt% Ru/C	0.1 M KOH	390	4
Mn oxide	0.1 M KOH	540	4
$Mn_3O_4/CoSe_2$	0.1 M KOH	450	5
NiFe-LDH/CNT	0.1 M KOH	308	6
NiFe-LDH/CNT	1 M KOH	247	6
BSCF[a]	0.1 M KOH	400	7

*Measured by Tafel plot. [a]The current density is 20 mA cm^{-2}.

Supplementary Table 2 Comparison of OER activities of NiFe/NF electrode with other reported catalysts using both GSA and ECAS in 1 M alkaline solutions.

Materials	j_{GSA}/mA cm^{2}	j_{ECAS}/mA cm^{2}	η/mV	Reference
NiFe/NF	300	6	300	This work
IrO_x	42	0.4	350	8
$NiCoO_x$	6	0.2	350	8
$NiFeO_x$	15	3	350	8

Supplementary Methods

Calculation of ECAS. The calculation of electrochemically active surface area (ECAS) is based on the measured double layer capacitance of the NiFe/NF electrode in 1 M KOH according to a previous published report.[8] Briefly, a potential range where no apparent Faradaic process happened was determined firstly using the static CV. The charging current i_c was measured from the CVs at different scan rates, as shown in Figure S10a. The relation between i_c, the scan rate (ν) and the double layer capacitance (C_{DL}) was given in eq 1.

$$i_c = \nu C_{DL} \quad (1)$$

Therefore, the slope of i_c as a function of ν will give a straight line with the slope equal to C_{DL} (Figure S10b). The C_{DL} of NiFe/NF measured from the scan rate dependent CVs is 1.10 mF.

For the estimation of ECAS, a specific capacitance (C_s) value C_s = 0.040 mF cm^{-2} in 1 M NaOH is adopted from previous reports.[8] As a result, the ECAS of the NiFe/NF is calculated to be 27.5 cm^2 according to eq 2.

$$ECAS = \frac{CDL}{Cs} \quad (2)$$

The geometric surface area (GSA) of the NiFe/NF electrode is 0.55 cm^2, therefore the roughness factor (RF) of as-prepared NiFe/NF electrode is 50 as determined by eq 3.

$$RF = \frac{ECAS}{GSA} \quad (3)$$

Supplementary References

1. Esswein AJ, McMurdo MJ, Ross PN, Bell AT, Tilley TD. Size-Dependent Activity of Co3O4 Nanoparticle Anodes for Alkaline Water Electrolysis. *J Phys Chem C* 2009, **113**(33)**:** 15068-15072.

2. Liang YY, Li YG, Wang HL, Zhou JG, Wang J, Regier T*, et al.* Co3O4 nanocrystals on graphene as a synergistic catalyst for oxygen reduction reaction. *Nat Mater* 2011, **10**(10)**:** 780-786.

3. Trotochaud L, Ranney JK, Williams KN, Boettcher SW. Solution-Cast Metal Oxide Thin Film Electrocatalysts for Oxygen Evolution. *J Am Chem Soc* 2012, **134**(41)**:** 17253-17261.

4. Gorlin Y, Jaramillo TF. A Bifunctional Nonprecious Metal Catalyst for Oxygen Reduction and Water Oxidation. *J Am Chem Soc* 2010, **132**(39)**:** 13612-13614.

5. Gao MR, Xu YF, Jiang J, Zheng YR, Yu SH. Water Oxidation Electrocatalyzed by an Efficient Mn3O4/CoSe2 Nanocomposite. *J Am Chem Soc* 2012, **134**(6)**:** 2930-2933.

6. Gong M, Li YG, Wang HL, Liang YY, Wu JZ, Zhou JG*, et al.* An Advanced Ni-Fe Layered Double Hydroxide Electrocatalyst for Water Oxidation. *J Am Chem Soc* 2013, **135**(23)**:** 8452-8455.

7. Suntivich J, May KJ, Gasteiger HA, Goodenough JB, Shao-Horn Y. A Perovskite Oxide Optimized for Oxygen Evolution Catalysis from Molecular Orbital Principles. *Science* 2011, **334**(6061)**:** 1383-1385.

8. McCrory CCL, Jung S, Peters JC, Jaramillo TF. Benchmarking Heterogeneous Electrocatalysts for the Oxygen Evolution Reaction. *J Am Chem Soc* 2013, **135:** 11.
